# Impaired hemodynamics of the patella in patients with patellofemoral pain: A case–control study

**DOI:** 10.1002/jeo2.12009

**Published:** 2024-02-28

**Authors:** Martin J. Ophey, Anne Westerweel, Maxime van Oort, Robert van den Berg, Gino M. M. J. Kerkhoffs, Igor J. R. Tak

**Affiliations:** ^1^ IJsveldFysio—Private Physical Therapy Clinic Nijmegen The Netherlands; ^2^ Department of Orthopaedic Surgery and Sports Medicine Amsterdam UMC, AMC location Amsterdam The Netherlands; ^3^ ESP Science and Education Vienna Austria; ^4^ Master Biomedical Sciences RU—Radboud University Nijmegen The Netherlands; ^5^ Physical Therapy Department FH Burgenland—University of Applied Science Pinkafeld Austria; ^6^ AIM—Austrian Institute of Management Advanced Physiotherapy & Management Eisenstadt Austria; ^7^ Amsterdam Collaboration on Health & Safety in Sports (ACHSS) IOC Research Center Amsterdam The Netherlands; ^8^ Academic Center for Evidence‐based Sports Medicine (ACES) Amsterdam The Netherlands; ^9^ Physiotherapy Utrecht Oost—Sports Rehabilitation and Manual Therapy Utrecht The Netherlands

**Keywords:** near‐infrared spectroscopy, patellofemoral pain

## Abstract

**Purpose:**

According to the homeostasis model, patellofemoral pain (PFP) arises as a consequence of disturbed homeostasis of anterior structures of the knee due to vascular insufficiency. Near‐infrared spectroscopy (NIRS) allows to measure changes of concentrations (µmol/cm^2^) of (de)‐oxygenated hemoglobine (HHb and O_2_Hb). The aim was to study differences in patellar hemodynamics between patients and healthy controls.

**Methods:**

Hemodynamics of patients (*n* = 30 [female = 20, age = 21.5, BMI = 22.9]) and controls (*n* = 30 (female = 18, age = 21.4, BMI = 22.4]) were evaluated for two activities (‘Prolonged Sitting’ and ‘Stair Descent’). Blinding for health status was implemented.

**Results:**

During ‘Prolonged Sitting’, PFP patients exhibited smaller decreases in mean changes for HHb (PFP [*M* = −1.5 to −1.9], healthy controls [*M* = −2.0 to −2.3]) and O_2_Hb (PFP [*M* = −2.0 to −3.2], healthy controls [*M* = −3.4 to −4.1]). However, these differences were statistically non‐significant (*p* = 0.14–0.82 and *p* = 0.056–0.18, respectively). Conversely, for ‘Stair Descent’, PFP patients showed statistically significant smaller decreases in mean changes for HHb (PFP [*M* = −1.9, SD = 1.8], healthy controls [*M* = −2.5, SD = 1.7], *p* = 0.043) and O_2_Hb (PFP [*M* = −3.2, SD = 3.2], healthy controls [*M* = −4.9, SD = 2.7], *p* = 0.004).

**Conclusions:**

The differences suggest potential impairment in patellar hemodynamics in PFP patients, providing support for the homeostasis model. Evidence‐based treatment strategies targeting patellar hemodynamics should be further refined and subjected to evaluation in clinical trials.

**Level of Evidence:**

Level III.

**Abbreviations**
AKPS: Anterior Knee Pain Scale
BMI: body mass index
CI: confidence interval
DPF: differential pathlength factor
DSDT: decline step‐down test
ES: effect size
HHb: deoxygenated hemoglobine
ISAK: International Society for the Advancement of Kinanthropometry
LLROM: lower limb range of motion
M: mean
MRI: magnetic resonance imaging
NIRS: near‐infrared spectroscopy
O_2_Hb: oxygenated hemoglobine
PFM: patellofemoral maltracking
PFP: patellofemoral pain
SD: standard deviation
SDC: smallest detectable change
VAS: Visual Analogue Scale

## BACKGROUND

The patellofemoral maltracking (PFM) model is a simultaneously acknowledged and debated model explaining the onset of patellofemoral pain (PFP) [36]. Patellar maltracking refers to altered kinematics of the patellofemoral joint, arising from compromised muscle function and impaired soft tissue flexibility [26, 41]. Methodological concerns regarding the validity of this pathomechanical model, originating in the 1980s, have been expressed recently [17, 25].

The homeostasis model represents an underexplored alternative model compared to the PFM model. According to this model, PFP arises as a consequence of the disturbance of homeostasis in both osseous and soft tissue within the peripatellar region due to supraphysiologic loading [12, 36]. Supporting evidence from observational studies comprises morphologic changes of the retinacula [24, 41], including neovascularisation and hyperinnervation [37, 38], and increased intraosseous water content and pressure of the patellar bone [20, 28]. Some authors propose that these changes are hypoxia‐induced due to peripatellar anastomotic ring vascular insufficiency [15, 37, 39, 42]. However, only a few clinical studies evaluated blood flow of the patella in PFP patients, suggesting reduced drainage time of the venous system, reduced pulsatile blood flow and statistically nonsignificant differences in blood perfusion [2, 19, 29].

Near‐infrared spectroscopy (NIRS), an optical, noninvasive method using a light‐source and ‐detector allows a continuous bone hemodynamics assessment [5, 27]. The patellar bone has never been studied with NIRS, but the results of a recent study indicate that NIRS measurements are sufficiently reliable to compare real‐time bone hemodynamics in PFP patients and healthy controls [34]. This opens a window of opportunity to evaluate if vascular insufficiency of the peripatellar anastomotic ring plays a role in PFP patients.

The objective of the current study was therefore to study potential differences in patellar hemodynamics between PFP patients and healthy controls in clinically relevant positions.

## METHODS

### Participants

This observational study was conducted according to the Declaration of Helsinki [46]. The study protocol was approved by the Medical Research Ethics Committee of Amsterdam UMC location University of Amsterdam (NL77408.018.21) and has been registered (ISRCTN 90377123) prior to the start of the data collection.

A convenient sample was recruited from (1) a private physical therapy clinic, and (2) the HAN University of Applied Sciences and (3) the Radboud University in Nijmegen, the Netherlands between February and May 2022. A senior physical therapist (MO) screened subjects based on inclusion and exclusion criteria (Table 1) through history taking and standardised physical examination.

**Table 1:** Criteria for inclusion and exclusion.

	Inclusion	Exclusion
General	Age: 18–40 years. Informed consent.	Previous or current clinical diagnosis of serious pathology (such as malignancy). Previous or current other clinical diagnosis of specific knee conditions (such as patellar instability or dislocation, jumpers knee, meniscus tears, or other ligament injury). Previous surgery (ankle, knee, hip, or lower back).
PFP patients	Pain: ∘ experienced around and/or behind the patella. ∘ aggravated by one or more of the following activities: Squatting, stair ambulation, jogging/running, hopping/jumping. ∘ lasting for three months or longer. ∘ not as a result of trauma. Experience worst pain levels of at least 3/10 on a Visual Analogue Scale (VAS‐W) during previous week.	Positive FABER/FADDIR (referred pain from the hip joint).
Healthy controls		Previous diagnosis of PFP. Complaints of ankle, knee, hip or lower back over the past 6 months requiring attention from a health care professional (physician, physical therapist), or resulting in missing more than one game, competition or training.

After inclusion, participants' demographic data including gender, age, body mass index (BMI), current smoking status and hours of sports per week were collected. Blood pressure was measured (Omron M6; Omron Healthcare) [1]. Furthermore, all participants completed the Tegner score and the Anterior Knee Pain Scale (AKPS) [23, 44]. The Tegner score evaluates the current physical activity level (0–10) [9, 21], with higher scores representing higher activity levels. The Dutch Tegner score is reliable (*ICC* = 0.93–0.97) and valid with an internal consistency of *r* = 0.73–0.83 [13]. The AKPS is a 13‐item questionnaire to quantify subjective symptoms and functional disabilities, with higher scores corresponding to fewer symptoms and disabilities [23]. The Dutch version of the AKPS is reliable (*ICC* = 0.98) and valid with an internal consistency of *r* = 0.78–0.80 [45]. Participants were instructed to cease any sports activity 12 h before the NIRS measurement.

### NIRS

Near‐infrared light penetrates human tissue superficially up to a depth of 4 cm [4]. The NIRS device used was the PortaLite (Artinis Medical Systems). The PortaLite is a continuous‐wave device and the sensor (13.4 cm^2^) consists of three light emitting diodes arranged at 30, 35 and 40 mm distance from the detector. The diodes transmit near‐infrared light at 760 and 820 nm. These wavelengths have specific absorption characteristics for deoxygenated (HHb) and oxygenated hemoglobine (O_2_Hb). Considering the NIRS measurement depth (roughly half the sensor‐detector distance [3]), the device assesses concentrations of HHb and O_2_Hb at depths ranging from 15 to 20 mm within the tissue. The assessment of HHb and O_2_Hb concentrations through NIRS relies on the adaptation of the Beer‐Lambert law, incorporating the ‘differential pathlength factor’ (DPF) [3, 11]. This adjustment is specifically designed to accommodate measurements conducted within biological tissue. Since no research with NIRS is done to examine patellar hemodynamics, the DPF was estimated with the following equation: $\frac{1}{2}{\left(\frac{3{\mu^{\prime }}_{s}\left(\lambda \right)}{\mu_{a}\left(\lambda \right)}\right)}^{1/2}$ which takes the absorption (*μ_a_*) and reduced scattering (*μ_s_*) coefficients of the human skull into account [40], resulting in a DPF of 8.66.

Data were sampled at a frequency of 10 Hz. Moreover, NIRS measures no absolute concentrations of HHb and O_2_Hb, but only relative changes of concentrations compared to a baseline. Therefore, all measurements were preceded by a 3‐min baseline and relative changes in HHb and O_2_Hb between baseline and experimental measurements (Δ) were calculated in μmol/cm^2^ [3]. The NIRS sensor was affixed to the skin with medical transparent double‐sided adhesive tape (2181 disks; 3M) and placed according to the standardised procedure as described previously [34]. The NIRS sensor was covered with an opaque cloth to reduce ambient light influence. Furthermore, the laboratory room had constant light and was maintained at a room temperature between 21 and 23°C.

### ‘Prolonged Sitting’ and ‘Stair Descent’

Patellar bone hemodynamics were evaluated during two clinically relevant activities for PFP patients: Prolonged sitting with the knees flexed and stair descent.

#### Activity 1 ‘Prolonged Sitting’

**Figure 1:**
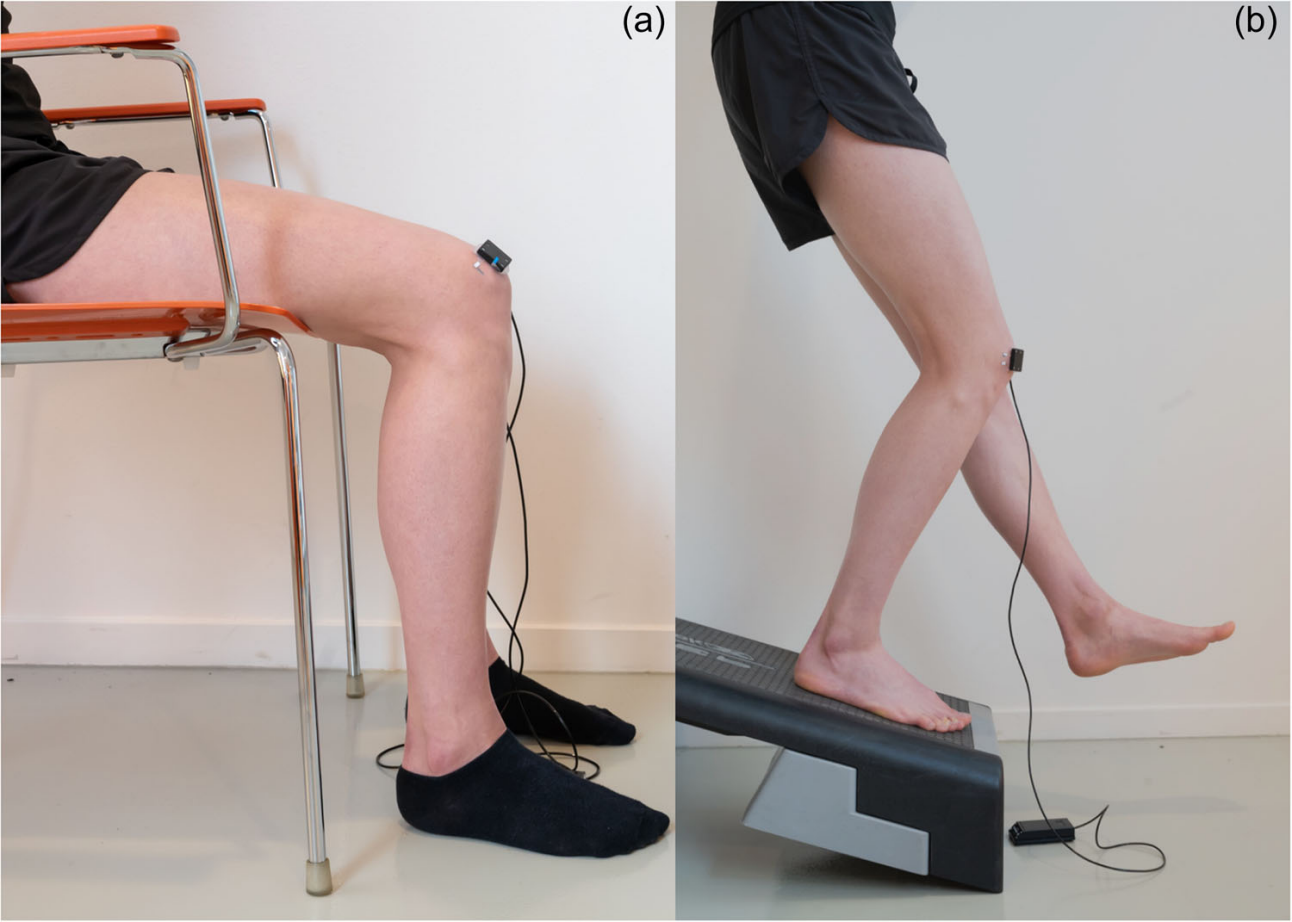
(a) Activity 1 ‘Prolonged Sitting’ and (b) activity 2 ‘Stair Descent’ (absence of the opaque cloth to visualise the set‐up, during measurements the sensor was covered).

After a 15‐min rest period where the participant's knees were flexed in 20 degrees, a 3‐min baseline was established in the same position, once stability of the NIRS signal was visually confirmed by examiner AW. Subsequently, the knees were flexed to 90 degrees for a period of 30 min (Figure 1a), and extended again to 20 degrees of flexion for 5 min. Accuracy of knee angles were evaluated with an extendable goniometer.

#### Activity 2 ‘Stair Descent’

Stair descent was replicated through the utilization of the previously developed Decline Step‐Down Test (DSDT) [32]. Following a 15‐min rest period in standing position and stable NIRS signal (visual inspection), a 3‐min baseline was established, while the participant was standing on the decline step‐down set‐up with extended knees. Then, the participant performed a motion simulating stair descent to 45 degrees of knee flexion and maintained this for 1 min (Figure 1b). Accuracy of this knee angle was evaluated with an extendable goniometer. After a 3‐min rest, the procedure was repeated for the other leg. The order of the starting leg was randomised, resulting in half of the participants started with the left leg and the others with the right leg.

Ophey et al. [34] reported moderate to almost perfect agreement of patellar NIRS measurements during Activity 1 ‘Prolonged Sitting’ [34]. For HHb concentrations, *ICCs* ranged from 0.51 to 0.75, with the smallest detectable change (*SDC*) ranging from 1.8 to 3.3. Similarly, for O_2_Hb concentrations, reported *ICCs* ranged from 0.56 to 0.95, with *SDCs* ranging from 0.6 to 1.5. These values are applicable to the 30 mm optode. Furthermore, Ophey et al. [34] noted moderate to substantial agreement during Activity 2 ‘Stair Descent’. For HHb concentrations, *ICC*s ranged from 0.50 to 0.68, with *SDCs* ranging from 0.5 to 1.0. For O_2_Hb concentrations, the reported *ICC*s ranged from 0.51 to 0.62, with *SDC*s ranging from 0.8 to 1.8. These values are pertinent to the 35 mm optode. It is important to highlight that the reliability of other optodes was found to be more variable, potentially attributed to the smaller sample size in the study by Ophey et al. [34]. Consequently, our decision was to exclusively analyse hemodynamics of the patellar bone using the aforementioned optodes.

### Secondary outcomes

#### Visual Analogue Scale (VAS)

The VAS offers a continuous scale (0–10) from ‘no pain’ to ‘extreme pain’, thereby providing a subjective pain assessment [22]. For Activity 1 ‘Prolonged Sitting’, pain levels were assessed at baseline (after 1.5 min), every two minutes while maintaining seating position with the knees flexed, and during recovery period (after two and four minutes). During Activity 2 ‘Stair Descent’, participants were queried about their pain level at baseline (after 1.5 min) and during the experimental measurement (after 0.5 min).

#### Patella skinfold and width

For a more comprehensive assessment of anthropometric characteristics of the anterior knee, prepatellar skinfold thickness and patella width were measured before placement of the NIRS sensor. Adherence to the guidelines of the International Society for the Advancement of Kinanthropometry (ISAK) was maintained for these anthropometric measurements [43]. Following training administered by a certified and ISAK (level 1) registered dietitian, skinfold thickness was measured at the centre of the patella using a skinfold caliper (Harpenden; Baty International), while patella width was measured at the broadest section of the patella using a slide caliper (Innovare; Cescorf). The execution of these measurements followed established protocols as previously detailed [34].

#### Lower limb range of motion (LLROM)

**Figure 2:**
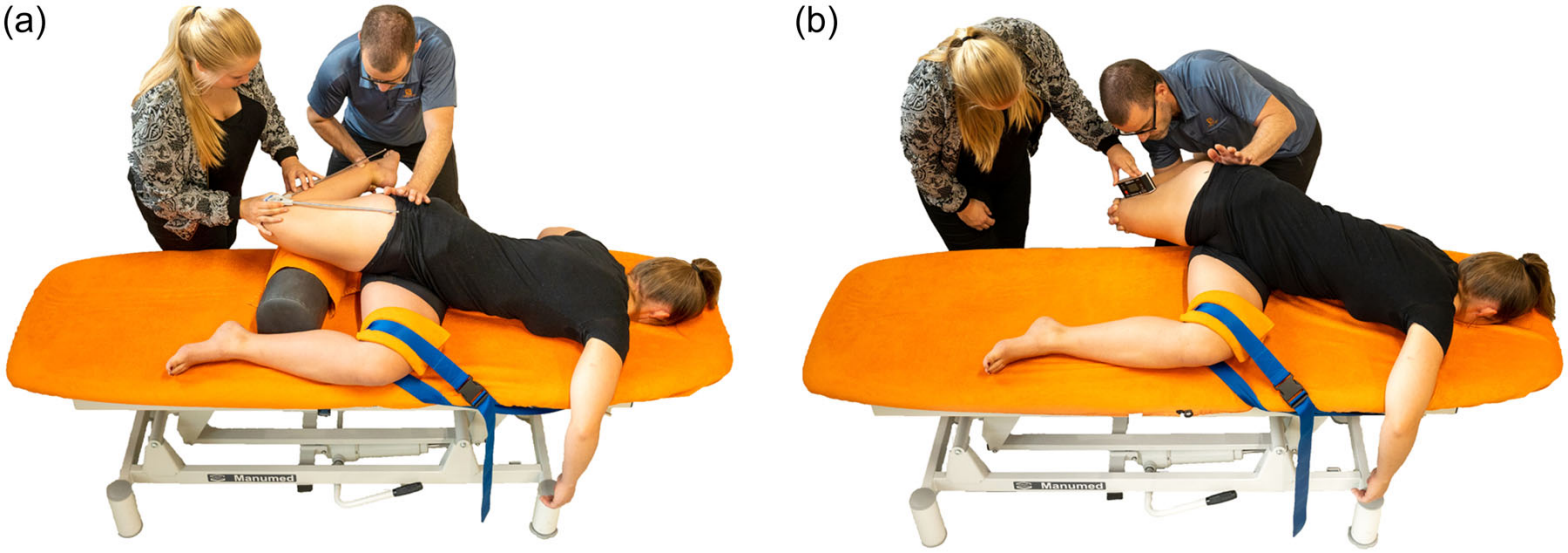
(a) Assessment of lower limb range of motion of knee flexion and (b) hip adduction (Ophey and colleagues).

The LLROM test assesses the flexibility of soft tissues over multiple joints within the kinetic chain [31]. Soft tissue flexibility of the anterior and lateral parts of the kinetic chain is evaluated by performing two passive movements [33], (1) maximal knee flexion with combined hip extension and upper body extension/rotation, lengthening the quadriceps and iliopsoas muscles (Figure 2a) and (2) maximal hip adduction with the same upper body position, lengthening the iliotibial tract, gluteal, abdominals and quadratus muscles in a combined fashion (Figure 2b). The cumulative score for total lower limb range of motion (total ROM) is derived by adding the scores for knee flexion and hip adduction. The LLROM test, previously described and established as reliable [31], was performed subsequent to the completion of NIRS measurements.

### Blinding

Blinding with respect for health status (PFP patient or healthy control) was implemented. Examiner MO was tasked with participant inclusion and exclusion. Examiner *AW* conducted the measurements without knowledge of the participant's health status.

### Sample size

Due to the absence of available literature on anticipated effect sizes in NIRS studies focusing on the hemodynamics of the patellar bone, our study adopted a pragmatic approach. The sample size was determined to be as large as possible within the constraints of our research group's available resources. We aimed to recruit a convenient sample comprising 30 individuals with PFP and 30 healthy controls for the study.

### Statistical analysis

The normality of data distribution was assessed through visual inspection and the Shapiro–Wilk test. Descriptive statistics, including means (*M*), standard deviations (SD) for continuous variables and percentages (%) for dichotomous variables, were employed to participants' baseline characteristics. PFP patients and healthy controls were not matched for any baseline characteristic. Continuous baseline characteristics were compared between PFP patients and healthy controls using student's *t* test (normally distributed data) or Mann–Whitney *U* test (not normally distributed data). Fisher's exact test was utilized for analysing differences in categorical baseline characteristics.

The NIRS sensor was placed on both knees of each participant, and knees were included separately for the statistical analysis. For PFP patients, only the symptomatic knees were included, while the nonsymptomatic knees were excluded from the statistical analysis. In contrast, for healthy controls, data from both knees were included in the analysis.

For Activity 1 ‘Prolonged Sitting’ four changes in HHb and O_2_Hb were assessed: (1) change between baseline and first 10 min of sitting with the knees flexed (ΔBas_Sit0‐10), (2) change between baseline and second 10 min of sitting with the knees flexed (ΔBas_Sit10‐20), (3) change between baseline and third 10 min of sitting with the knees flexed (ΔBas_Sit20‐30), (4) change between third 10 min of sitting with the knees flexed and five minutes of recovery time (ΔSit20‐30_Rec). For Activity 2 ‘Stair Descent’, the change in HHb and O_2_Hb between baseline and 45 degrees of knee flexion (ΔBas_Step) was assessed. Mean and standard deviation in HHb and O_2_Hb changes between baseline and experimental measurement were calculated for the 30 mm optode during Activity 1 ‘Prolonged Sitting’ and the 35 mm optode during Activity 2 ‘Stair Descent’. The first and last three seconds were removed to mitigate the influence of movement artifacts in the data.

Differences between PFP patients and healthy controls were analysed using student's *t* test (normally distributed data) or Mann–Whitney *U* test (not normally distributed data). Additionally, the changes of HHb and O_2_Hb were visualised with plots. Effect size (*ES*) was calculated using Cohen's *d* to present the magnitude of the change between baseline and experimental measurement, with an *ES* of 0.2 considered small, 0.5 medium and ≥0.8 large [7].

The NIRS measurements were conducted using Oxysoft, version 3.2.72 (Artinis). Subsequently, the Oxysoft files underwent conversion into MATLAB files (R2020a version 9.8.0.1323502; Mathworks). In MATLAB, a moving average filter was applied to the raw values, and changes between baseline and experimental measurements were calculated. The resulting outcomes were then entered into Excel (Microsoft Office version 16). Statistical analyses were carried out using SPSS (version 28.0) with a significance level set at *p* < 0.05.

## RESULTS

**Figure 3:**
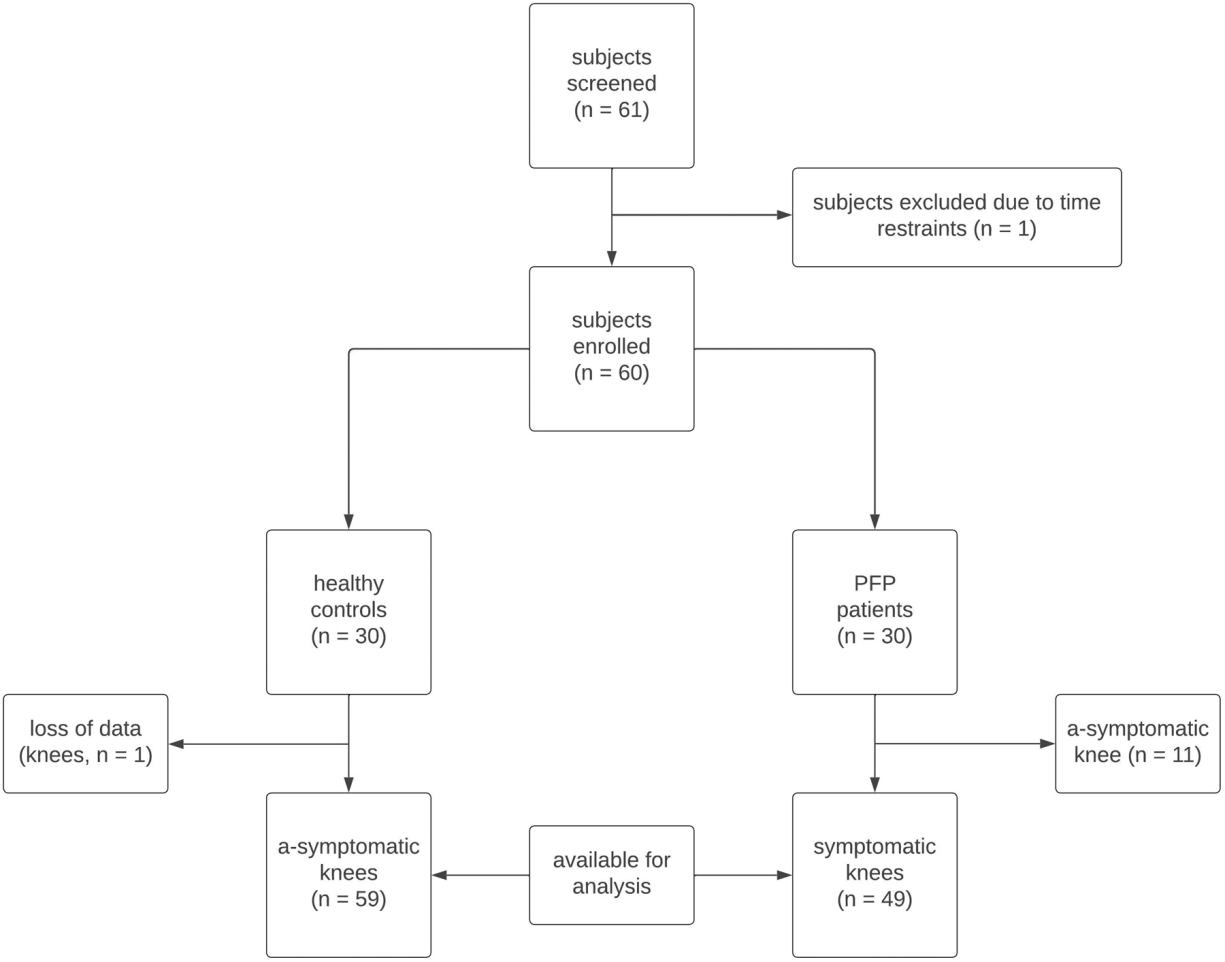
Flowchart of the inclusion process.

A total of 61 subjects underwent screening for participation, with sixty ultimately meeting the inclusion criteria. Unfortunately, one subject had to be excluded due to personal time constraints (Figure 3 flowchart). All participants successfully completed both Activity 1 ‘Prolonged Sitting’, and Activity 2 ‘Stair Descent’ as outlined in the study protocol. However, technical issues with the PortaLite device resulted in the loss data for one knee of a healthy control.

Table 2 presents the baseline characteristics of all participants. The cohort comprised 30 PFP patients (20 females [66.7%], mean age 21.5 years [SD = 2.4], mean BMI 22.9 kg/m^2^ [SD = 3.2]) and 30 healthy controls (18 females [60.0%], mean age 21.4 years [SD = 2.5], mean BMI 22.4 kg/m^2^ [SD = 2.3]). Patients with PFP exhibited statistically significant lower Tegner scores (*t*[58] = 2.69; *p* = 0.009, *d* = 0.73) and AKPS (*t*[31] = 10.44; *p* < 0.001, *d* = 2.69) compared to healthy controls, confirming correct group allocation.

**Table 2:** Baseline characteristics.

Characteristics	PFP patients	Healthy controls
Participants, *n*	30	30
Female, *n* (%)	20 (66.7)	18 (60.0)
Age (years)	21.5 (2.4)	21.4 (2.5)
BMI (kg/m^2^)	22.9 (3.2)	22.4 (2.3)
Bilateral PFP, *n* (%)	19 (63.3)	n/a
Symptom duration (mos)	47 (38.2)	n/a
VAS‐W (0–10)	5 (1.5)	n/a
Smoking, *n* (%)	5 (16.7)	1 (3.3)
Blood pressure (mm/Hg)		
Systolic	124.9 (14.1)	120.1 (11.6)
Diastolic	80.5 (11.1)	78.5 (8.2)
Sport participation (hours per week)	4.2 (2.4)	5.7 (5.1)
Tegner (0–10)	5.4 (1.6)**	6.4 (1.1)**
AKPS (0–100)	82.5 (8.6)***	99.2 (1.7)***
Patella (mm)		
Skinfold	9.1 (4.6)*	7.5 (1.8)*
Width	50.7 (4.5)	52.1 (3.6)
LLROM (deg)		
Knee flexion	100.5 (10.4)***	114.6 (10.4)***
Hip adduction	27.1 (7.6)***	31.3 (3.3)***
Total ROM	127.5 (19.0)***	145.9 (11.6)***

*Note*: Data are numbers (percentages), mean (standard deviation) or median (interquartile range 25%–75%).

Abbreviations: AKPS, anterior knee pain scale; BMI, body mass index in kilogram bodyweight per m^2^; deg, degrees; LLROM, lower limb range of motion; mm, millimetre; mmHg, millimetre of mercury; mos, months; *n*, number; n/a, not applicable; VAS‐W, visual analogue scale for worst pain.

* *p* < 0.05

** *p* < 0.01

*** *p* < 0.001.

### Activity 1 ‘Prolonged Sitting’

**Figure 4:**
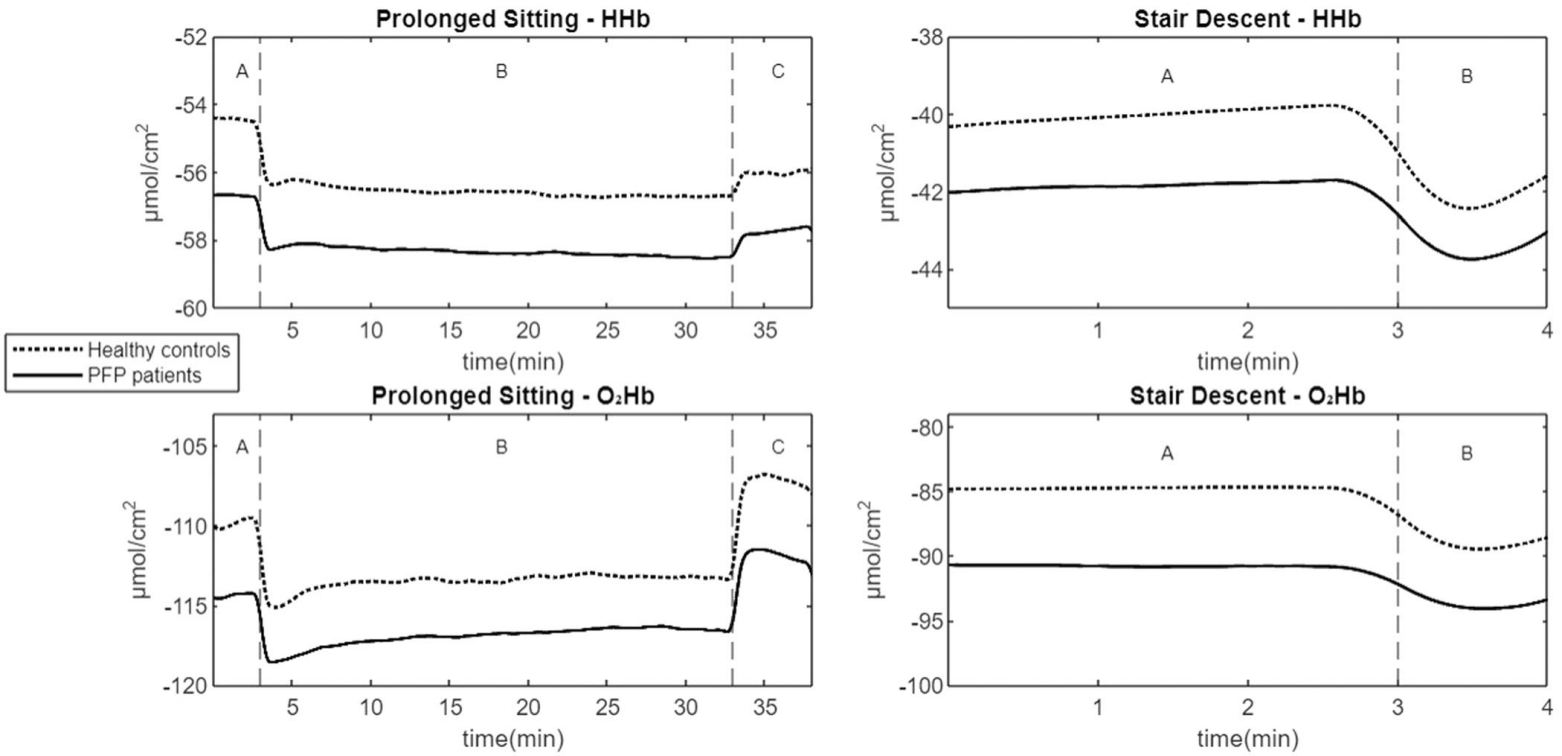
Plotted means of HHb and O_2_Hb (in µmol/cm^2^) during Activity 1 ‘Prolonged Sitting’ and Activity 2 ‘Stair Descent’. (A) baseline (3 min); (B) ‘Prolonged Sitting’ (30 min) or ‘Stair Descent’ (1 min), respectively; (C) recovery time after ‘Prolonged Sitting’ (5 min). HHb, deoxygenated hemoglobine; m, min; O_2_Hb, oxygenated hemoglobine.

While PFP patients exhibited smaller decreases in mean changes of HHb and O_2_Hb in PFP patients compared to healthy controls during Activity 1 ‘Prolonged Sitting’, these differences between groups were not statistically significant for all parameters, with *p* values ranging from 0.14 to 0.82 and from 0.06 to 0.18, respectively (Table 3). Plotted means of HHb and O_2_Hb during Activity 1 ‘Prolonged Sitting’ are presented in Figure 4.

**Table 3:** Changes in HHb and O_2_Hb (in µmol/cm^2^) during activity 1 ‘Prolonged Sitting’ and activity 2 ‘Stair Descent’.

	HHb				O_2_Hb			
Activity 1 ‘Prolonged Sitting’	PFP patients mean (SD)	Healthy controls mean (SD)	*t* Value (*df*)	*p* Value *ES*	PFP patients mean (SD)	Healthy controls Mean (*SD*)	*t* Value (*df*)	*p* Value *ES*
ΔBas_Sit0‐10^a^	−1.5 (1.4)	−2.0 (2.1)	−1.48 (103)	*p* = 0.14	−3.8 (2.4)	−4.1 (3.6)	−1.36 (106)	*p* = 0.18
ΔBas_Sit10‐20^b^	−1.7 (1.7)	−2.2 (2.2)	−1.41 (106)	*p* = 0.16	−2.4 (3.5)	−3.6 (3.7)	−1.66 (106)	*p* = 0.10
ΔBas_Sit20‐30^c^	−1.8 (1.8)	−2.3 (2.2)	−1.31 (106)	*p* = 0.19	−2.0 (3.5)	−3.4 (3.7)	−1.94 (106)	*p* = 0.06
ΔSit20‐30_Rec^d^	0.7 (1.2)	0.7 (1.4)	−0.23 (104)	*p* = 0.82	4.6 (4.8)	5.9 (4.8)	1.43 (104)	*p* = 0.16
Activity 2 ‘Stair Descent’								
ΔBas_Step^e^	−1.9 (1.8)	−2.5 (1.7)	−2.05 (106)	*p* = 0.043; *d* = 0.39*	−3.2 (3.2)	−4.9 (2.7)	−2.95 (106)	*p* = 0.004; *d* = 0.57**

Abbreviations: *df*, degrees of freedom; ES, effect size (Cohens ‘d’); HHb, deoxygenated hemoglobine; O_2_Hb, oxygenated hemoglobine; SD, standard deviation.

^a^ Change between baseline and first 10 min of sitting with the knees flexed.

^b^ Change between baseline and second 10 min of sitting with the knees flexed.

^c^ Change between baseline and third 10 min of sitting with the knees flexed.

^d^ Change between third 10 min of sitting with the knees flexed and 5 min of recovery time.

^e^ Change between baseline and 45 degrees of knee flexion.

* *p* < 0.05

** *p* < 0.01.

### Activity 2 ‘Stair Descent’

The decreases in mean changes of HHb and O_2_Hb in PFP patients were statistically significantly smaller than those in healthy controls, with (*t*[106] = −2.05; *p* = 0.043, *d* = 0.39) and (*t*[106] = −2.95; *p* = 0.004, *d* = 0.57), respectively (Table 3). Plotted means of HHb and O_2_Hb during Activity 2 ‘Stair Descent’ are illustrated in Figure 4.

### Secondary outcomes

**Figure 5:**
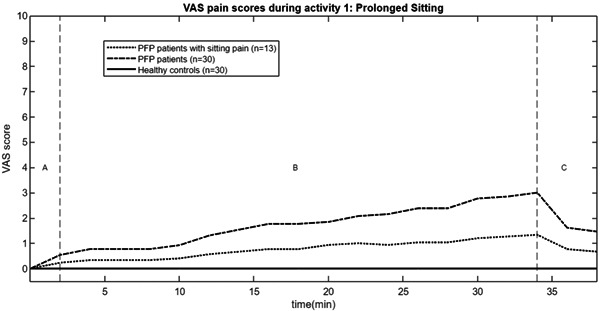
VAS during activity 1 ‘Prolonged Sitting’.

Healthy controls experienced no pain during both activities. In Activity 1 ‘Prolonged Sitting’, 13 (43%) PFP patients reported some knee pain, with a mean time to onset of nine minutes (SD = 9.0) (Figure 5). During Activity 2 ‘Stair Descent’, 25 (83%) PFP patients reported some knee pain, with a mean VAS of 3/10 (SD = 1.8). This difference was statistically significant compared to healthy controls (*t*[29] = −7.75; *p* < 0.01, *d* = 2.00). Additionally, PFP patients had statistically significant thicker prepatellar skinfold (*t*[61] = −2.28; *p* = 0.026, *d* = 0.46). Furthermore, PFP patients demonstrated statistically significant lower soft tissue flexibility of the lower limb in knee flexion (*t*[79] = 5.44; *p* < 0.001, *d* = 1.10), hip adduction (*t*[62] = 3.59; *p* < 0.001, *d* = 0.75) and total ROM (*t*[74] = 5.83; *p* < 0.001, *d* = 1.12) (Table 2).

## DISCUSSION

This was the first study to evaluate hemodynamics of the patella in positions clinically relevant for PFP patients. The main finding was smaller decreases in concentrations of (de‐)oxygenated hemoglobine (HHb and O_2_Hb) of the patellar bone during Activity 2 ‘Stair Descent’ among PFP patients when compared to healthy controls. This suggests a potential impairment in hemodynamics of the patella in PFP patients, and opens up a new window of opportunities to develop more effective evidence‐based treatment strategies for PFP patients.

In the current study, nearly half (43%) of PFP patients reported experiencing some knee pain during Activity 1 ‘Prolonged Sitting’. This aligns with previous reports indicating that 55% of PFP patients encountered ‘problems with prolonged sitting’, as assessed by item 8 of the AKPS [10]. The current study is the first to evaluate patellar bone hemodynamics during an extended sitting period. Although decreases in concentrations of HHb and O_2_Hb were observed to be smaller in PFP patients during Activity 1 ‘Prolonged Sitting’, the between‐group comparisons did not reach statistical significance, possibly due to insufficient power. Näslund et al. [29] found a reduction in patellar bone blood flow in PFP patients measured by photoplethysmography (PPG) during 5 min of knee flexion in a supine position. Since PPG measures the amplitude of pulsatile blood flow in bone tissue based on attenuation changes in the reflected signal [27], it is biologically plausible that a reduced amplitude in the PPG signal corresponds to smaller decreases in concentrations of HHb and O_2_Hb when evaluated with NIRS.

This study marked the first real‐time monitoring of pain during prolonged sitting in PFP patients using a VAS. As the sitting period extended, an increasing number of patients began reporting knee pain, with pain intensity gradually escalating during sitting (Figure 5). Interestingly, following the initial decrease in the first 10 min of sitting with the knees flexed, concentrations of HHb and O_2_Hb also showed a gradual increase during the sitting period (Figure 4). This lends support to the vascular insufficiency theory and suggests the presence of a homeostatic pain mechanism as an explanation for the occurrence of sitting pain in PFP patients [36].

In the current study, a significant majority (83%) of PFP patients reported pain during Activity 2 ‘Stair Descent’, aligning with the previously reported 88% of PFP patients experiencing pain during stair ambulation [35]. The simulated stair descent in this activity induces an eccentric action of the quadriceps muscles, involving simultaneous contraction and stretch, leading to increased patellofemoral contact forces [6]. Notably, the current study is the first to investigate patellar bone hemodynamics during active loading of the patellofemoral joint. Arnoldi [2] hypothesised that quadriceps muscle contraction amplifies compression at the base of the patella, impacting venous outflow of the patella's anastomotic ring. The absence of quadriceps muscle contraction during Activity 1 ‘Prolonged Sitting’ may result in less vascular insufficiency, potentially explaining fewer differences in patellar hemodynamics between PFP patients and healthy controls during this activity.

Given the cross‐sectional nature of the current study, it is not possible to draw conclusions about impaired patellar hemodynamics as a cause for the onset of PFP. However, the association between impaired hemodynamics and ongoing anterior knee pain in PFP patients appears plausible. A previous study observed an increase in HHb and O_2_Hb concentrations of the patella during venous occlusion with a thigh cuff in healthy controls [34]. Consequently, smaller decreases in HHb and O_2_Hb concentrations in symptomatic knees of PFP patients (as identified during activity 2 ‘Stair Descent’) might be linked to such venous occlusion. In essence, a reduced decrease in concentrations of (de‐)oxygenated hemoglobine may signify venous occlusion of the peripatellar anastomotic ring in PFP patients.

The posterior surface of the apex patellae serves as the most important exit point of the venous system in distal posterior direction, draining into the saphenous and popliteal vein [2]. Additionally, clusters of veins exit the basis patellae in proximal direction, with veins leaving the anterior, medial and lateral aspect of the patella also documented [2]. We hypothesise that venous outflow is compromised not only by quadriceps muscle contraction but also by lower soft tissue flexibility. This aligns with Arnoldi's early 1990s proposition [2], where he discussed reduced venous outflow as a potential pain mechanism of PFP, involving ‘extraosseous compression by the skin and subcutaneous tissue during stretching of these structures’. In the current study, we observed lower soft tissue flexibility (LLROM) in PFP patients, and interventions targeting LLROM in PFP patients led to an immediate reduction of pain and disability in the short‐term [33].

Van der Heijden et al. [19] investigated patellar bone perfusion using dynamic contrast‐enhanced magnetic resonance imaging (MRI) in PFP patients and healthy controls. This MRI investigation was conducted with the participant's knees in extension and unloaded in a supine position. This specific positioning could account for their observation of no differences in patellar bone perfusion between PFP patients and healthy controls.

The observed prepatellar skinfold thickness in PFP patients and healthy controls was 9.1 and 7.5 mm, respectively, resulting in skin thickness (skinfold divided by two) of 4.6 and 3.8 mm, respectively. When the superficial layer (e.g., skin) is less than 4 mm, it has a nonsignificant confounding contribution on NIRS measurements [14]. Only when the superficial layer exceeds 13 mm does it predominantly influence the measurements [14]. Since the reported thickness of the skin of both PFP patients and healthy controls was close to or below the recommended 4 mm, we conclude that skin thickness did not significantly affect our measurements. Additionally, the difference in prepatellar skinfold thickness between PFP patients and healthy controls was statistically significant. PFP patients had a lower Tegner score, participated in sports fewer hours per week, and had a higher BMI, although the differences of the latter two were not statistically significant. Thus, one explanation of the observed difference in skinfold thickness may be a reduced activity pattern in PFP patients with associated changes in body composition. Although lower activity levels and increased BMI of PFP patients have been reported in the literature [16, 18], the design of the current study does not allow for causal inferences. Additionally, the skin thickness difference between the two groups was <1 mm, which may (at least partially) have been the result of measurement error. Before the experiments, we were trained according to the ISAK criteria, and for measurements, we used the high‐quality Harpenden caliper. Thus, we aimed to minimise measurement variability. Given that the observed difference between both groups was <1 mm, and skin compression does not affect NIRS measurements [34], we assumed that this difference did not affect the NIRS measurement in the current study.

Näslund et al. [30] demonstrated an increase in patellar bone blood flow during contraction of the quadriceps muscle in healthy participants. Given that contraction of the quadriceps muscle increases patellar bone blood flow in healthy individuals and venous outflow is decreased in PFP patients, the commonly recommended quadriceps‐oriented exercise therapy [8] to reduce PFM may potentially exacerbate symptoms of PFP, as reported by many clinicians. Näslund et al. [30] also showed that intermittent contractions (with 2 s of rest between repetitions) reduce patellar bone blood flow in healthy participants. Therefore, we propose that a multimodal treatment strategy, considering impaired hemodynamics of the patella, should include interventions to improve LLROM involving soft tissue, hip‐oriented exercises, intermittent quadriceps‐oriented exercise therapy and education on homeostatic pain mechanisms. Assessing the impact of such a multimodal treatment strategy on the improvement of patellar hemodynamics and its effectiveness in reducing pain and disability should be explored in future research.

We acknowledge certain limitations in the current study. The pragmatic enrolment of participants, recruiting 30 PFP patients and 30 healthy controls, may have led to an inappropriate sample size. Specifically, for Activity 1 ‘Prolonged Sitting’, the failure to detect statistically significant differences may be attributed to only 43% of PFP patients experiencing pain during prolonged sitting. A post hoc power analysis indicated that a sample size of *n* = 110 for each group would be needed to reach a power of 0.80. Therefore, future investigations on patellar hemodynamics, especially those assessing pain during prolonged sitting, should consider defining pain during this activity as an inclusion criterion and aim to increase the number of participants accordingly. The findings from the current study can serve as a basis for calculating sample sizes in future research.

We acknowledge another limitation related to the design of Activity 2 ‘Stair Descent’. While this activity was intended to simulate descending stairs, the necessity of a 1‐min quadriceps muscle contraction in 45 degrees of knee flexion emerged as a requirement for generating reproducible NIRS measurements. Unfortunately, this aspect diminishes the validity of the set‐up in mimicking a real‐world situation of descending stairs. Given the sensitivity of the NIRS device employed in the current study, it is improbable that a more dynamic simulation of stair descent would yield NIRS data of satisfactory quality.

## CONCLUSIONS

The current study indicates potential impairment in the hemodynamics of the patella in PFP patients when compared to healthy controls. The findings provide support for the homeostasis model as a contributing factor to the existence of PFP, possibly attributed to vascular insufficiency in the peripatellar anastomotic ring. Recommendations for evidence‐based treatment strategies targeting the hemodynamics of the patella should be further refined and subjected to evaluation in clinical trials.

## AUTHOR CONTRIBUTIONS

Martin J. Ophey, Robert van den Berg, Igor J.R. Tak and Gino M.M.J. Kerkhoffs conceived the study. Martin J. Ophey, Anne Westerweel, Maxime van Oort, Robert van den Berg, Igor J.R. Tak and Gino M.M.J. Kerkhoffs were responsible for the study design and analysis. Martin J. Ophey, Anne Westerweel, Maxime van Oort and Robert van den Berg were responsible for data collection. All authors have made substantial revisions to earlier drafts and approved the final manuscript.

## CONFLICT OF INTEREST STATEMENT

The authors declare no conflict of interest.

## ETHICS STATEMENT

By the Medical Research Ethics Committee of Amsterdam UMC location University of Amsterdam under number NL77408.018.21, The Netherlands. ISRCTN Trial Registration under number: 90377123. Written informed consent procedure according to the Medical Research Involving Human Subjects Act.

## Data Availability

Research data will be available upon reasonable request.

## References

[1] Altunkan Ş , Ilman N , Kayatürk N , Altunkan E . Validation of the Omron M6 (HEM‐7001‐E) upper‐arm blood pressure measuring device according to the International Protocol in adults and obese adults. Blood Press Monitor. 2007;12:219–225. 10.1097/MBP.0b013e3280f813d0 17625394

[2] Arnoldi CC . Patellar pain. Acta Orthop Scand. 1991;62:1–29. 10.3109/17453679109153923 1882690

[3] Artinis Medical Systems . Manual Portalite for Oxysoft 3.0.95 and higher—version 1610. 2016. Manual Portalite for Oxysoft 3.0.95 and higher—version 1610. 2016.

[4] Aziz SM , Khambatta F , Vaithianathan T , Thomas JC , Clark JM , Marshall R . A near infrared instrument to monitor relative hemoglobin concentrations of human bone tissue in vitro and in vivo. Rev Sci Instrum. 2010;81:043111. 10.1063/1.3398450 20441329

[5] Binzoni T , van de Ville D . Noninvasive probing of the neurovascular system in human bone/bone marrow using near‐infrared light. J Innovative Opt Health Sci. 2011;04:183–189. 10.1142/S1793545811001447

[6] Brechter JH , Powers CM . Patellofemoral joint stress during stair ascent and descent in persons with and without patellofemoral pain. Gait Posture. 2002;16:115–123. 10.1016/S0966-6362(02)00090-5 12297253

[7] Cohen J . Statistical power analysis for the behavioral sciences. 2nd ed. Hillsdale, NJ: Lawrence Erlbaum Associates; 1988.

[8] Collins NJ , Barton CJ , van Middelkoop M , Callaghan MJ , Rathleff MS , Vicenzino BT , et al. 2018 Consensus statement on exercise therapy and physical interventions (orthoses, taping and manual therapy) to treat patellofemoral pain: recommendations from the 5th International Patellofemoral Pain Research Retreat, Gold Coast, Australia, 2017. Br J Sports Med. 2018;52:1170–1178. 10.1136/bjsports-2018-099397 29925502

[9] Collins NJ , Misra D , Felson DT , Crossley K , Roos E . Measures of knee function: International Knee Documentation Committee (IKDC) Subjective Knee Evaluation Form, Knee Injury and Osteoarthritis Outcome Score (KOOS), Knee Injury and Osteoarthritis Outcome Score Physical Function Short Form (KOOS‐PS), Knee Ou. Arthritis Care Res. 2011;63(Suppl 1):S208–S228. 10.1002/acr.20632 PMC4336550 22588746

[10] Collins NJ , Vicenzino B , van der Heijden RA , van Middelkoop M . Pain during prolonged sitting is a common problem in persons with patellofemoral pain. J Orthop Sports Phys Ther. 2016;46:658–663. 27374012 10.2519/jospt.2016.6470

[11] Delpy DT , Cope M , Zee P , Arridge S , Wray S , Wyatt J . Estimation of optical pathlength through tissue from direct time of flight measurement. Phys Med Biol. 1988;33:1433–1442. 10.1088/0031-9155/33/12/008 3237772

[12] Dye SF . The pathophysiology of patellofemoral pain: a tissue homeostasis perspective. Clin Orthop Relat Res. 2005;436:100–110. 10.1097/01.blo.0000172303.74414.7d 15995427

[13] Eshuis R , Lentjes GW , Tegner Y , Wolterbeek N , Veen MR . Dutch translation and cross‐cultural adaptation of the lysholm score and tegner activity scale for patients with anterior cruciate ligament injuries. J Orthop Sports Phys Ther. 2016;46:976–983. 27681449 10.2519/jospt.2016.6566

[14] Franceschini MA , Fantini S , Paunescu LA , Maier JS , Gratton E . Influence of a superficial layer in the quantitative spectroscopic study of strongly scattering media. Appl Opt. 1998;37:7447–7458. 10.1364/AO.37.007447 18301579

[15] Fulkerson JP . The etiology of patellofemoral pain in young, active patients: a prospective study. Clin Orthop Relat Res. 1983;179:129–133. 10.1097/00003086-198310000-00018 6617004

[16] Glaviano NR , Baellow A , Saliba S . Physical activity levels in individuals with and without patellofemoral pain. Phys Ther Sport. 2017;27:12–16. 10.1016/j.ptsp.2017.07.002 28780340

[17] Grant C , Fick CN , Welsh J , McConnell J , Sheehan F . A word of caution for future studies in patellofemoral pain: a systematic review with meta‐analysis. Am J Sports Med. 2020;49:539–551. 10.1177/0363546520926448 PMC9906796 32816535

[18] Hart HF , Barton CJ , Khan KM , Riel H , Crossley KM . Is body mass index associated with patellofemoral pain and patellofemoral osteoarthritis? A systematic review and meta‐regression and analysis. Br J Sports Med. 2017;51:781–790. 10.1136/bjsports-2016-096768 27927675

[19] van der Heijden RA , Poot DHJ , Ekinci M , Kotek G , van Veldhoven PLJ , Klein S , et al. Blood perfusion of patellar bone measured by dynamic contrast‐enhanced MRI in patients with patellofemoral pain: a case‐control study. J Magn Reson Imaging. 2018;48:1344–1350. 10.1002/jmri.26174 29734499 PMC6221059

[20] Ho K‐Y , Hu HH , Colletti PM , Powers CM . Running‐induced patellofemoral pain fluctuates with changes in patella water content. Eur J Sport Sci. 2014;14:628–634. 10.1080/17461391.2013.862872 24283889

[21] Howe TE , Dawson LJ , Syme G , Duncan L , Reid J . Evaluation of outcome measures for use in clinical practice for adults with musculoskeletal conditions of the knee: a systematic review. Man Ther. 2012;17:100–118. 10.1016/j.math.2011.07.002 21872524

[22] Kahl C , Cleland JA . Visual analogue scale, numeric pain rating scale and the McGill pain Questionnaire: an overview of psychometric properties. Phys Ther Rev. 2005;10:123–128. 10.1179/108331905X55776

[23] Kujala UM , Jaakkola LH , Koskinen SK , Taimela S , Hurme M , Nelimarkka O . Scoring of patellofemoral disorders. Arthrosc‐J Arthrosc Relat Surg. 1993;9:159–163. 10.1016/s0749-8063(05)80366-4 8461073

[24] Lack S , Anthony L , Noake J , Brennan K , Zhang B , Morrissey D . Medial and lateral patellofemoral joint retinaculum thickness in people with patellofemoral pain: a case‐control study. J Ultrasound Med. 2019;38:1483–1490. 10.1002/jum.14828 30251436

[25] Lack S , Neal B , De Oliveira Silva D , Barton C . How to manage patellofemoral pain—understanding the multifactorial nature and treatment options. Physical Therapy in Sport. 2018;32:155–166. 10.1016/j.ptsp.2018.04.010 29793124

[26] Lankhorst NE , Bierma‐Zeinstra SMA , Van Middelkoop M . Risk factors for patellofemoral pain syndrome: a systematic review. J Orthop Sports Phys Ther. 2012;42:81–A12. 22031622 10.2519/jospt.2012.3803

[27] Meertens R , Casanova F , Knapp KM , Thorn C , Strain WD . Use of near‐infrared systems for investigations of hemodynamics in human in vivo bone tissue: a systematic review. J Orthop Res. 2018;36:2595–2603. 10.1002/jor.24035 29727022

[28] Miltner O , Siebert CH , Schneider U , Niethard FU , Graf J . Patellar hypertension syndrome in adolescence: a three‐year follow up. Arch Orthop Trauma Surg. 2003;123:455–459. 10.1007/s00402-003-0564-2 12910357

[29] Näslund J , Walden M , Lindberg LG . Decreased pulsatile blood flow in the patella in patellofemoral pain syndrome. Am J Sports Med. 2007;35:1668–1673. 10.1177/0363546507303115 17567822

[30] Näslund JE , Näslund S , Lundeberg E , Lindberg LG , Lund I . Bone blood flow is influenced by muscle contractions. J Biomed Sci Eng. 2011;04:490–496. 10.4236/jbise.2011.47062

[31] Ophey MJ , Bennink D , Bernsen JE , Blazevic I , van Bergen R , van den Berg R , et al. Patients with patellofemoral pain have lower soft tissue flexibility of the kinetic chain compared to healthy controls: a case‐control study. J Bodyw Mov Ther. 2023;36:203–209. 10.1016/j.jbmt.2023.06.006 37949561

[32] Ophey MJ , Bosch K , Khalfallah FZ , Wijnands AMMP , van den Berg RB , Bernards NTM , et al. The decline step‐down test measuring the maximum pain‐free flexion angle: a reliable and valid performance test in patients with patellofemoral pain. Physical Therapy in Sport. 2019;36:43–50. 10.1016/j.ptsp.2018.12.007 30641448

[33] Ophey MJ , Crooijmans GAHM , Frieling SMW , Kardos D , van den Berg R , Kerkhoffs G , et al. Short‐term effectiveness of an intervention targeting lower limb range of motion on pain and disability in patellofemoral pain patients: a randomized, non‐concurrent multiple‐baseline study. J Bodyw Mov Ther. 2021;26:300–308. 10.1016/j.jbmt.2020.12.028 33992263

[34] Ophey MJ , Westerweel A , van Oort M , van den Berg R , Kerkhoffs GMMJ , Tak IJR . Near‐infrared spectroscopy measurements are reliable for studying patellar bone hemodynamics and affected by venous occlusion, but not by skin compression. J Exp Orthop. 2023;10(1):124. 10.1186/s40634-023-00709-6 38017345 PMC10684445

[35] Post WR , Fulkerson J . Knee pain diagrams: correlation with physical examination findings in patients with anterior knee pain. Arthrosc‐J Arthrosc Relat Surg. 1994;10:618–623. 10.1016/s0749-8063(05)80058-1 7880352

[36] Sanchis‐Alfonso V , Ramírez‐Fuentes C , Roselló‐Sastre E , Dye S , Teitge R . Pathophysiology of anterior knee pain. In: Dejour D , Zaffagnini S , Arendt EA , Sillanpää P , Dirisamer F , editors. Patellofemoral pain, instability, and arthritis. Berlin: Springer Berlin Heidelberg; 2020. p. 93–116.

[37] Sanchis‐Alfonso V , Roselló‐Sastre E . Immunohistochemical analysis for neural markers of the lateral retinaculum in patients with isolated symptomatic patellofemoral malalignment. Am J Sports Med. 2000;28:725–731. 10.1177/03635465000280051801 11032232

[38] Sanchis‐Alfonso V , Roselló‐Sastre E , Monteagudo‐Castro C , Esquerdo J . Quantitative analysis of nerve changes in the lateral retinaculum in patients with isolated symptomatic patellofemoral malalignment. Am J Sports Med. 1998;26:703–709. 10.1177/03635465980260051701 9784819

[39] Sanchis‐Alfonso V , Roselló‐Sastre E , Revert F , García A . Histologic retinacular changes associated with ischemia in painful patellofemoral malalignment. Orthopedics. 2005;28:593–599. 10.3928/0147-7447-20050601-16 16138473

[40] Scholkmann F , Wolf M . General equation for the differential pathlength factor of the frontal human head depending on wavelength and age. J Biomed Opt. 2013;18:105004. 10.1117/1.JBO.18.10.105004 24121731

[41] Schoots EJM , Tak IJR , Veenstra BJ , Krebbers YMJ , Bax JG . Ultrasound characteristics of the lateral retinaculum in 10 patients with patellofemoral pain syndrome compared to healthy controls. J Bodyw Mov Ther. 2013;17:523–529. 10.1016/j.jbmt.2013.03.005 24139014

[42] Selfe J , Harper L , Pedersen I , Breen‐Turner J , Waring J , Stevens D . Cold legs: a potential indicator of negative outcome in the rehabilitation of patients with patellofemoral pain syndrome. Knee. 2003;10:139–143. 10.1016/S0968-0160(02)00085-6 12787996

[43] da Silva VS , Vieira MFS . International Society for the Advancement of Kinanthropometry (ISAK) Global: international accreditation scheme of the competent anthropometrist. Rev Bras de Cineantropometria e Desempenho Hum. 2020;22:1–6. 10.1590/1980-0037.2020v22e70517

[44] Tegner Y , Lysholm J . Rating systems in the evaluation of knee ligament injuries. Clin Orthop Relat Res. 1985;198:42–49. 10.1097/00003086-198509000-00007 4028566

[45] Ummels PEJ , Lenssen AF , Barendrecht M , Beurskens AJHM . Reliability of the Dutch translation of the Kujala Patellofemoral Score Questionnaire. Physiother Res Int. 2017;22:e1649. 10.1002/pri.1649 26308151

[46] World Medical Association . World Medical Association Declaration of Helsinki: ethical principles for medical research involving human subjects. JAMA. 2013;310:2191–2194. 10.1001/jama.2013.281053 24141714

